# Accumulated Hard-tissue Debris Removal Using Different Ultrasonic Power Settings: A Micro-CT Study

**DOI:** 10.1055/s-0045-1810441

**Published:** 2025-08-07

**Authors:** Flávia de Moura Pereira, Kusai Baroudi, Tarun Walia, Vivek Padmanabhan, Emmanuel Joao Nogueira Leal da Silva, Carolina de Oliveira Lima, Rafael Pino Vitti, Rayana Duarte Khoury, Flávia Goulart da Rosa Cardoso

**Affiliations:** 1 Division of Endodontics, Department of Odontology, University of Taubaté – UNITAU, Taubaté, SP, Brazil; 2 Department of Clinical Sciences, College of Dentistry, Ajman University, Ajman, United Arab Emirates; 3 Centre of Medical and Bio-allied Health Sciences Research, Ajman, United Arab Emirates; 4 Department of Pediatric and Preventive Dentistry, RAK College of Dental Sciences, RAK Medical and Health Sciences University, Ras Al Khaimah, United Arab Emirates; 5 Department of Endodontics – Grande Rio University (UNIGRANRIO), Duque de Caxias, Rio de Janeiro, RJ, Brazil; 6 Department of Endodontics, Rio de Janeiro Federal University, Rio de Janeiro, RJ, Brazil; 7 Department of Restorative Dentistry, São Paulo State University (Unesp), Institute of Science and Technology, São José dos Campos, Brazil

**Keywords:** passive ultrasonic activation, hard-tissue debris, micro–computed tomography

## Abstract

**Objectives:**

To evaluate the effectiveness of the Irrisonic Power ultrasonic tip activated at different power settings (10 and 30%) on the removal of accumulated hard-tissue debris (AHTD) from mandibular premolars using micro-computed tomography (micro-CT) analysis.

**Materials and Methods:**

A total of 20 extracted mandibular premolars were pair-matched based on root canal morphology using micro-CT imaging. All specimens were prepared with Reciproc R25 and randomly assigned to two experimental groups ( *n* =  10) according to the power setting used during final irrigation: Irrisonic Power 10% or Irrisonic Power 30%. Supplementary irrigation was performed with sodium hypochlorite and EDTA. AHTD was quantified pre- and post-irrigation using standardized micro-CT analysis and ImageJ software.

**Statistical Analysis:**

Data were statistically analyzed using the Mann–Whitney test ( *α* =  0.05).

**Results:**

Both protocols significantly reduced AHTD after supplementary irrigation. The Irrisonic Power 30% group exhibited lower residual debris (0.16  ±   0.13%) than the Irrisonic Power 10% group (0.90  ±   1.16%), with a statistically significant difference ( *p* <  0.05).

**Conclusion:**

Supplementary irrigation with the Irrisonic ultrasonic tip at 30% power was more effective in removing AHTD than the 10% power setting.

## Introduction

During root canal preparation, auxiliary chemical solutions act as disinfectants and lubricants. They help eliminate pulp tissue, necrotic remnants, and debris produced by the mechanical action of instruments. 1 However, accumulated hard-tissue debris (AHTD) may persist within the root canal system (RCS). This is clinically relevant, as AHTD can harbor microorganisms, especially in difficult-to-access areas, compromising disinfection protocols and interfering with root canal filling procedures. 2 3 4

Studies have shown that conventional syringe and needle irrigation may be ineffective in removing AHTD, especially in anatomical irregularities and/or difficult-to-reach areas. 5 6 To overcome this limitation, several strategies to optimize the irrigating solution activity have been proposed, such as the use of different types of needle, sonic activation, passive ultrasonic irrigation (PUI), and the use of a negative pressure irrigation system. 7 8 9 10 PUI is the technique used to activate the root canal irrigant using ultrasonically oscillating noncutting tips as a supplementary protocol after the completion of biomechanical preparation. The acoustic streaming provided by the ultrasonic activation allows a deeper penetration of irrigants into complex anatomic regions of the RCS. 11 The efficacy of PUI in AHTD removal has been studied extensively. 7 12 13 14 15 16 17 18 Although studies show an improvement in AHTD removal after PUI protocols, none of them have shown that this technique was able to completely clean and disinfect the entire root canal space. 17

Irrisonic tip (Helse Dental Technology, São Paulo, Brazil) is an instrument introduced to be used after root canal instrumentation, during PUI. Due to the inherent fragility of its small core size (tip size #25, 0.01 taper), it is recommended to be activated at a power of 10%. 19 Irrisonic Power tip (Helse Dental Technology) is a recently developed instrument launched aiming to overcome the limited activation power of its predecessor. This instrument presents some modifications in the insert length and bending angle, which according to the manufacturer allows this device to be used at higher powers without major risks of fracture. Since the activation power of an instrument is directly related to its cleaning capacity, 8 20 the use of this new tip with higher activation power could lead to a greater AHTD removal, improving the root canal cleaning during the final irrigation protocol.

Compared with other activation systems reported in the literature, such as EndoActivator, EndoUltra, and XP-Endo Finisher, the Irrisonic Power tip presents notable structural and functional differences. The EndoActivator operates at lower sonic frequencies, generating limited cavitation and acoustic streaming effects. 8 20 The EndoUltra, although ultrasonic, is battery-powered and may present limitations in continuous activation and tip flexibility. 7 17 The XP-Endo Finisher, although effective in mechanical agitation, relies on direct contact with canal walls and shape-memory alloy expansion to clean irregular anatomies, which may limit its efficacy in narrow or complex regions. 17 18 In contrast, the Irrisonic Power tip was developed with a reinforced core and modified geometry, specifically in its insert length and bending angle, which allow safe use at higher ultrasonic power settings without increasing the risk of tip deformation or fracture. 19 These structural modifications enhance the stability and resistance of the insert, enabling more intense and sustained acoustic streaming, which may improve irrigant penetration and AHTD removal, especially in the apical third and anatomically complex areas of the RCS. 8 17 18

In this context, the aim of the present study was to evaluate the effectiveness of the Irrisonic Power ultrasonic tip activated using 10 and 30% power settings on the removal of AHTD from mandibular premolars using micro–computed tomography (micro-CT) analysis. The null hypothesis tested was that there would be no difference in AHTD removal when different powers are used to activate the Irrisonic Power insert.

## Materials and Methods

### Sample Size Calculation

Sample size was calculated from previous studies with similar methodologies. 13 17 21 The effect size for this study was 0.91 and added to a power β = 95% with a statistical significance level set at α = 5% in an F test family for one-way analysis (G*Power 3.1.7 Software for MacBook, Heinrich Heine, Universität Düsseldorf). The ideal total sample size required to observe significant differences was indicated to be 17 specimens. A total of 20 specimens ( *n* = 10) were used to compensate for possible sample loss.

### Sample Selection

This study was approved by the Local Ethics Committee (#2.975.186). A total of 55 human lower premolars were selected and stored in 0.1% thymol solution at 5°C. Digital radiographs were taken for each specimen in the mesiodistal and buccolingual direction to verify the internal anatomy of the root canals. Digital periapical radiographs were taken using an X-ray unit (Gnatus Timex 70E, Ribeirão Preto, SP, Brazil) operating at 70 kVp, 8 mA, and an exposure time of 0.2 seconds. A digital CMOS sensor (RVG 5200, Carestream Dental, Atlanta, GA, USA) was used for image acquisition. The X-ray tube was positioned at a 30-cm distance from the sensor, and all images were captured with a paralleling technique to ensure standardization. These radiographs were used to confirm the presence of a single canal, and the absence of previous root canal treatment, calcifications, root resorptions, or fractures. Initially, 55 extracted human mandibular premolars were scanned using micro-CT. From this pool, 20 teeth were selected based on morphological similarity in terms of length, canal volume, and three-dimensional anatomy, to ensure group homogeneity.

The teeth were scanned on a microtomograph (SkyScan 1174, Bruker, Kontich, Belgium) with the following acquisition parameters: 800 mA and 50 kV, 27 µm isotropic resolution, 0.5 mm thick aluminum filter, 5,200 millisecond exposure time, rotation step of 0.5, and 180 degrees about the vertical axis. All images were reconstructed using NRecon software (v1.6.1.0; Bruker, Kontich, Belgium) with the same parameters: ring artifact reduction, 8 beam hardening correction (35%), and smoothing 4 for all images. To ensure sample homogeneity, the surface area (mm ^2^ ) and volume (mm ^3^ ) of root canals were calculated by the CTAn program (Bruker Micro-CT, Kontich, Belgium) and the three-dimensional image was obtained with CTVol (Bruker Micro-CT, Kontich, Belgium). Subsequently, the selected teeth were randomly distributed into two experimental groups ( *n* = 10). Normal distribution of the data ( *ρ* > 0.05; Shapiro-Wilk) and group homogeneity were evaluated for morphological dimensions (length, volume, and three-dimensional anatomy), confirming the anatomical similarity between experimental groups ( *ρ* > 0.05; Student's t-test and Mann-Whitney test).

### Root Canal Preparation

After access cavity preparation, a glide path was created by scouting a stainless-steel size #15 K-file (Dentsply Maillefer, Ballaigues, Switzerland) up to the working length (WL), which was established as being 1 mm short of the apical foramen. The apices of the teeth were sealed with hot glue to create a close-end system. Then root canals were prepared using Reciproc R25 (25/.08v) instrument (VDW, Munich, Germany) in a reciprocating motion (RECIPROC ALL) powered by an electric motor (VDW Silver; VDW). The instrument was moved apically using a sequence of three in-and-out reciprocating motions, with approximately 3 mm of amplitude and slight apical pressure. Throughout instrumentation and after preparing each third (cervical, middle, and apical), the root canals were irrigated using with 5 mL of 2.5% NaOCl using a 30-G NaviTip needle (Ultradent Products Inc, South Jordan, USA) using a total of 15 mL to each specimen. The foraminal patency was maintained using the #15 K-file during the entire procedure. The specimens were submitted to new micro-CT procedures, applying the above-mentioned parameters, to calculate the root canal space volume and AHTD after preparation.

### Supplementary Irrigation Protocol

**Fig. 1:**
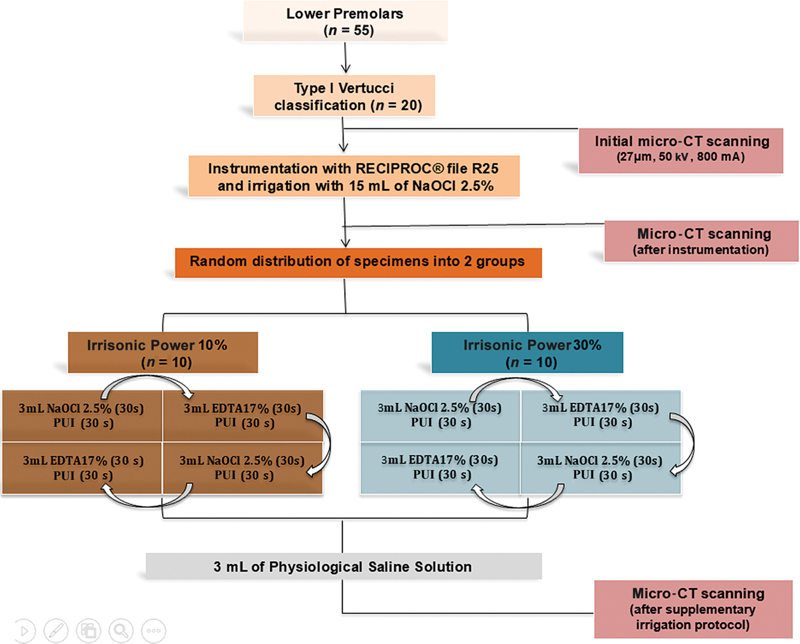
Flowchart of experimental procedures. PUI, passive ultrasonic irrigation.

One tooth from each pair-matched teeth was randomly assigned to one of the two experimental groups ( *n* = 10), according to the final irrigation protocol:

- Irrisonic Power 10%: Root canals were irrigated with 3 mL 2.5% NaOCl using a NaviTip needle, held within the root canal for 30 s, and then agitated for 30 s using the Irrissonic Power tip 1 mm short of the WL mounted on a piezoelectric ultrasonic device (P5 Newton XS; Acteon Satelec, Norwich, UK), with the power setting at 10% (2/20). Next, the root canals were irrigated with 3 mL of 17% EDTA, held within the root canal for 30 s, and then activated for 30 s using the same technique as described above. This protocol was repeated twice, and a final rinse with 3 mL of saline solution was performed ( Fig. 1 ).
- Irrisonic Power 30%: Root canals were irrigated with 3 mL 2.5% NaOCl using a NaviTip needle, held within the root canal for 30 s, and then agitated for 30 s using the Irrissonic Power tip 1 mm short of the WL mounted on a piezoelectric ultrasonic device (P5 Newton XS; Acteon Satelec, Norwich, UK), with the power setting at 30% (6/20). Next, the root canals were irrigated with 3 mL of 17% EDTA, held within the root canal for 30 s, and then activated for 30 s using the same technique as described above. This protocol was repeated twice, and a final rinse with 3 mL of saline solution was performed ( Fig. 1 ).

The same final volume of irrigating solution (15 mL) was used in each group, being 6 mL 2.5% NaOCl, 6 mL 17% EDTA, and 3 mL saline solution. Finally, the specimens were dried with absorbent paper points (Dentsply Maillefer). A single experienced operator conducted all experimental procedures. At the end of this stage, the specimens were submitted to new micro-CT procedures, applying the above-mentioned parameters.

### Micro-CT Scanning and Three-Dimensional Analysis

After supplementary irrigation protocols, the tooth image slices were recorded with their respective postoperative images from the 3D Slicer 4.4.0 software (available at http://www.slicer.org ), overlapping the images at an accuracy greater than 1 voxel. 17 Root canal volume, before and after instrumentation, was calculated by the ImageJ program (v.1.49, Fiji, Madison, WI). 21 Material with dentin-like density located in the region of the instrumented root canal was considered AHTD, 13 which was quantified, as previously described, by the intersection between images before and after irrigation activation protocols 21 and expressed as the percentage of the total volume of root canals after instrumentation of each sample.

For the quantification of dentinal debris, the reconstructed axial images were exported in TIFF format and analyzed using ImageJ. All images were converted to 8-bit grayscale and binarized using the Otsu automatic thresholding method, with a fixed threshold range set between 35 and 255 gray values. This range was selected based on preliminary evaluations to distinguish high-density debris from the surrounding canal space. Following binarization, images were converted to binary, with debris appearing as high-intensity (white) structures. Small particles and background noise were excluded using the “Analyze Particles” tool with a minimum size threshold of 50 pixels. For each cross-sectional image, the area occupied by debris was measured and expressed as a percentage relative to the total area of the root canal lumen. This procedure was applied uniformly to all samples and groups to ensure consistency and comparability of measurements. Then the images obtained after the quantification of dentinal debris were transformed into three-dimensional images using the CTVol program (v. 2.2.1, Bruker microCT).

### Statistical Analysis

The volume (mm ^3^ ) and accumulation of dentinal debris (%) of supplemental irrigation protocols were used as reference parameters to verify if specimens within groups had similar conditions. Volume demonstrated a normal distribution of data (Shapiro-Wilk test— *ρ* > 0.05) and therefore the Student's *t* -test was used to compare volume between groups. For the evaluation of debris (%), the sample did not present normal distribution, and therefore the nonparametric Mann-Whitney test was performed ( *α* = 0.05).

## Results

The baseline homogeneity of the experimental groups was confirmed with respect to root canal length, volume, surface area, and initial AHTD volume after root canal preparation ( *ρ* >  0.05). Table 1 presents the pre- and post-irrigation AHTD volumes and their respective percentages for both groups. Both the supplemental cleaning protocols significantly reduced AHTD compared with the baseline (paired *t* -test, *ρ* < 0.05). However, the Irrisonic Power 30% group achieved a significantly greater reduction in AHTD (mean final value: 0.16%) than the Irrisonic Power 10% group (mean final value: 0.90%) (Mann-Whitney test, *ρ* <  0.05).

**Table 1:** Mean and standard deviation of the volume of the root canal space and accumulated debris after instrumentation and supplementary irrigation protocols

Parameters		Irrisonic Power 10%	Irrisonic Power 30%	
		(mean ± standard deviation)			
Root canal space volume	Post-instrumentation (mm ^3^ )	9.08 ± 2.91	9.70 ± 2.40
	Post-activation (mm ^3^ )	9.55 ± 2.97		10.66 ± 2.48
Debris volume	Post-activation (mm ^3^ )	0.08 ± 0.09	0.01 ± 0.01
	Post-activation (%)	0.90 ± 1.16		0.16 ± 0.13

**Fig. 2:**
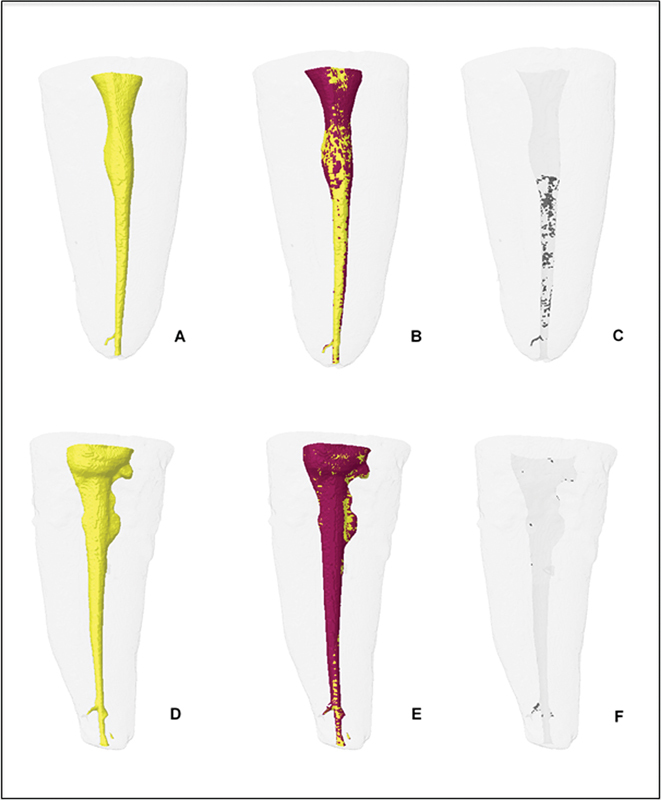
Representation of three-dimensional models of flattened lower premolar canals. Irrisonic Power 10% group: ( **A** ) Post-instrumentation volume (yellow), ( **B** ) volume before (yellow) and post-activation (red), and ( **C** ) remnant debris accumulation (gray). Irrisonic Power 30% group: ( **D** ) Post-instrumentation volume (yellow), ( **E** ) volume before (yellow) and post-activation (red), and ( **F** ) remnant debris accumulation (gray).

Fig. 2 shows a three-dimensional representation of mandibular premolars before and after the supplementary irrigation protocol using the Irrisonic Power insert at 10 and 30% power setting.

## Discussion

The AHTD produced during mechanical preparation are considered a clinically relevant issue, since it may be packed into anatomical complexities harboring bacterial contents away from the disinfection procedures. 14 16 22 The amount of hard-tissue debris accumulated after biomechanical preparation has been extensively demonstrated and discussed, pointing out the need of a final irrigation protocol after root canal preparation. 1 2 3 4

The present study evaluated the efficacy of different ultrasonic powers in the use of PUI supplemental cleaning protocols in the removal of AHTD from uniradicular mandibular premolars by micro-CT. This three-dimensional nondestructive technique allows the quantitative and qualitative evaluation of AHTD accumulation in RCS irregularities and has been used successfully as a gold standard technique to evaluate this. 13 22 23

Due to the heterogeneity found in the morphology of root canals and to avoid anatomic biases that may interfere with the study outcomes, specimens were pre-screened using micro-CT. The micro-CT screening of the length, area, and volume, and three-dimensional configuration was performed to provide an overall anatomical mapping of the root canals. 13 21 Based on these data and on root canal configuration and morphology, 10 pair-matched mandibular premolars were grouped and further allocated into one of the two experimental groups. Statistical analysis confirmed the effective balance among the groups with respect to the baseline parameters, thus enhancing the internal validity of this study.

The results of the present study showed that supplementary root canal cleaning using passive ultrasonic agitation improves the efficiency of removal of AHTD. These results are in line with previous studies that also showed similar AHTD removal using PUI. 6 16 24 However, in the present study Irrisonic Power 30% group had higher root canal cleaning capacity when compared with the Irrisonic Power 10% group ( *ρ* < 0.05). Therefore, the null hypothesis tested was rejected. In the present study, supplemental irrigation protocols were identical, differing only in the power setting of the equipment. Consequently, the results can be explained by a more precise activation of the irrigating solution and an increased acoustic flow 24 provided by the high power employed (30%) through the ultrasonic device. Possibly, these mechanical improvements in the instrument design allowed this newly developed tip to be used at 30% power level with better results than the same tip used at 10% power level. However, to the best of the authors' knowledge, this is the first study evaluating the removal of AHTD with Irrisonic Power tips.

Although both the final irrigation protocols showed a significant decrease in the accumulation of hard-tissue debris ( *ρ* < 0.05), none of them was able to completely remove the AHTD. This finding is in agreement with several previous studies, 6 13 16 17 18 24 25 and highlights the fact that the biomechanical preparation of RCS invariably creates accumulation of AHTD in areas of anatomical irregularities that cannot be completely removed with the currently available techniques.

This study presents some limitations that should be acknowledged. First, only uniradicular mandibular premolars were included, which typically present relatively simple and straight canal anatomies. As such, the results may not be fully generalizable to molars or other teeth with more complex canal systems, such as those containing curvatures, isthmuses, or accessory canals. Second, the absence of additional control groups using other activation systems or a no-activation protocol limits the ability to make broader comparative conclusions. Future studies should include a wider variety of tooth anatomies and additional control groups to validate and expand upon the present findings.

Although both the power settings resulted in a significant reduction of AHTD, the 30% power group demonstrated a substantially lower final debris percentage. This difference, although numerically modest, may be clinically relevant, especially in anatomical conditions such as isthmuses, apical deltas, or oval-shaped canals, where debris tends to persist even after thorough instrumentation and irrigation. 6 15 17 The ability to activate irrigants more intensely, while maintaining structural safety, could enhance cleaning and disinfection in these challenging areas. Previous studies have shown that improved irrigant activation can significantly increase debris removal from complex root canal regions. 7 16 18 These findings support the consideration of higher ultrasonic activation powers in clinical protocols and suggest that future investigations should evaluate the performance of the Irrisonic Power tip in multi-rooted teeth or anatomically irregular canals to validate its effectiveness across different clinical scenarios.

Within the conditions of the present study, it can be concluded that both the supplementary irrigation protocols were effective in removing AHTD. However, Irrisonic Power at 30% power level has been shown to be more efficient at removing debris from root canals.

## Conclusion

Both supplementary irrigation protocols using the Irrisonic Power tip significantly reduced the volume of accumulated hard-tissue debris in mandibular premolars. However, activation at 30% power proved to be significantly more effective than the 10% power setting.
